# A species-specific tale of quantitative disease resistance (QDR) in tomato

**DOI:** 10.1093/plcell/koaf263

**Published:** 2025-11-05

**Authors:** Pei Qin Ng

**Affiliations:** Assistant Features Editor, The Plant Cell, American Society of Plant Biologists; Department of Plant Sciences, University of Cambridge, Cambridge CB2 3EA, UK

Due to the molecular complexity and inefficacy of the gene-for-gene immune response, it is a considerable challenge to engineer disease-resistant crops against necrotrophic pathogens (Mengiste and Liao 2025). Quantitative disease resistance (QDR) is an alternative to the “all or nothing” gene-for-gene model in plants, in which QDR confers plants partial resistance to a broad spectrum of pathogens. Therefore, enhancing QDR in crop plants is critical to provide resistance against necrotrophic pathogens. Recent work by Delplace et al. (2025; highlighted by Ng [2025]) explored QDR diversity among *Arabidopsis* accessions, identifying core QDR genes involved in response to infection by the necrotrophic fungus *Sclerotinia sclerotiorum*.

In their new work, Severin Einspanier and colleagues (Einspanier et al. 2025) inspected the transcriptome of 5 tomato species to characterize the evolution of QDR regulation in response to *S. sclerotiorum*. Previously, the authors used a detached leaf assay to describe a diversity of resistance phenotypes among 4 wild tomato species (also used in the current study), with none showing 100% resistance to *S. sclerotiorum* (Einspanier et al. 2024). Before proceeding into in-depth transcriptomic analysis, the authors profiled the phylogenetic relationship of the 5 tomato species using protein sequence data. Lesion growth rate analysis revealed vastly different susceptibilities of respective cultivars. Through differential gene expression analysis, the authors concluded that the number of differentially expressed genes (DEGs) does not explain the QDR variability, as there were fewer DEGs for resistant versus susceptible *S. lycopersicoides* and vice versa for *S. pennelli*. The authors reported a total of 239 core DEG orthologues between the resistant and susceptible genotypes of all 5 tomato species, which followed diverse expression patterns upon *S. sclerotium* infection.

To understand the influence of gene network rewiring in QDR responses in susceptible versus resistant cultivars, the authors deployed weighted gene correlation network analysis on 2 species: *S lycopersicoides* and *S. pennellii*. This revealed clear transcriptional differences between susceptible and resistant genotypes, allowing the authors to describe a 2-tier QDR system comprised of a broad functioning defence network and species-specific responses (Figure). The analysis also showed that the shared genes from resistance modules possessed a lower-than-average transcript diversification index, indicating signs of purifying selection. The authors hypothesized that QDR regulation in *S. lyocopersicoides* and *S. pennellii* might therefore be driven by differential regulation (“rewiring”) of a relatively well-conserved, ancestral suite of genes.

The authors then performed an integrative analysis of weighted gene correlation network analysis, directed gene regulatory networks, and differential expression to identify central regulators of QDR in all tested species. These central regulators are often transcription factors forming the core of the transcription network (Sorrells and Johnson 2015). Interestingly, the NAC family TF *NAC29* was identified as a key gene in mediating QDR responses toward *S. sclerotiorum*. The NAC transcription factors (TFs) are key players in plant developmental processes, such as embryogenesis, leaf senescence, and root development (Wen et al. 2022; Xiong et al. 2025). In this study, the role of NAC29 in QDR is unique to *S. pennellii*, while the same gene is not associated with QDR in the other species. In a highly susceptible genotype of *S. pennellii*, *NAC29* contained a premature stop codon within the coding sequence, suggesting a new functional role of *NAC29* in QDR resistance phenotype.

In summary, the work of Einspanier and colleagues on tomato presents early evidence of gene network rewiring in QDR against a necrotrophic pathogen, as well as the co-option of a TF in driving QDR through the species-specific case study of *S. penelli*. Future direction of this study will require generating a *nac29* knockout in the *S. penelli*–resistant cultivar to confirm its role in *S. sclerotiorum* disease resistance. This demonstrates the complex gene regulation involved in plant disease resistance mechanisms via QDR and opens more avenues to our understanding of the evolution driving this defence mechanism.

**Figure.**
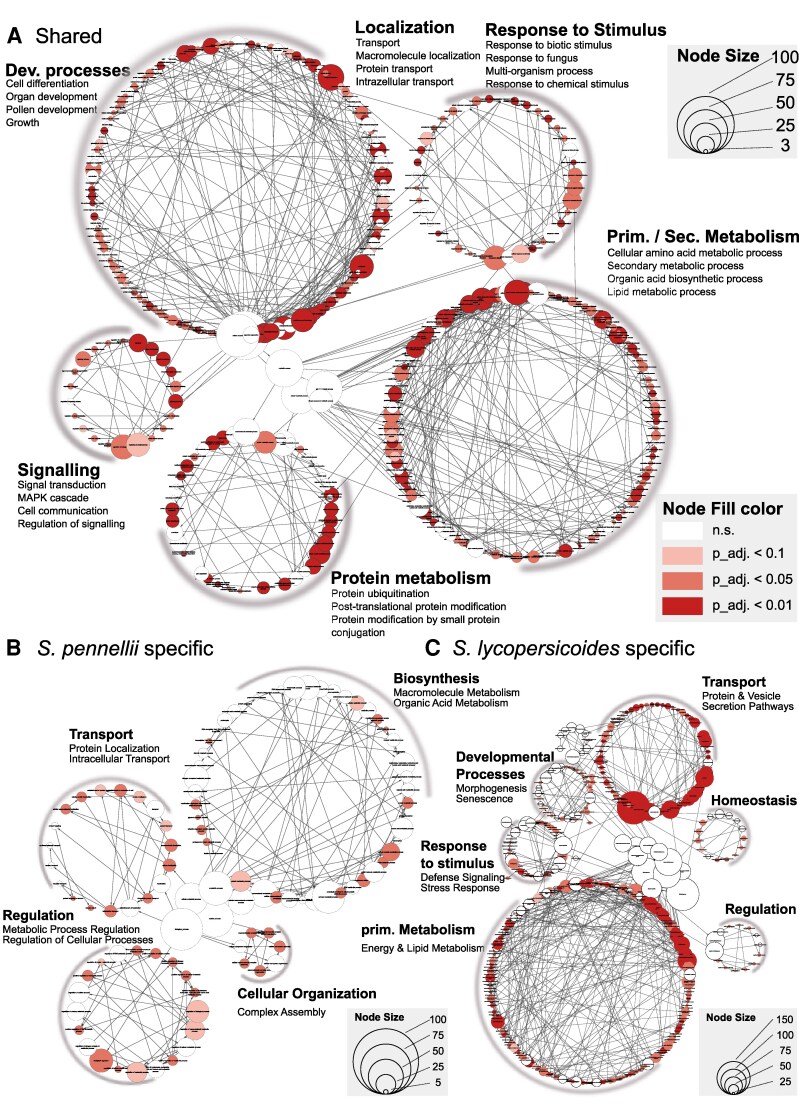
Gene ontology (GO) network analysis showing processes shared between the resistance genotypes of *S. pennelli* and *S. lyocopersicoides*  **(A)** and unique to the resistance genotype of the two species **B**, **C)**, respectively. Reprinted from Einspanier et al. (2025), Figure 7.

## Recent related articles in *The Plant Cell*:

- Delplace et al. (2025) explored QDR toward *Sclerotinia sclerotiorum* in *Arabidopsis thaliana* accessions.
- Yue et al. (2024) used a genome-wide association study to identify a NAC TF variant associated with fruit ripening in apple.

## Data Availability

No new data were generated or analysed in support of this article.

## Dive Curated Terms

The following phenotypic, genotypic, and functional terms are of significance to the work described in this paper:

NAC CHEBI: CHEBI:7421
